# A Systems Analysis With “Simplified Source-Sink Model” Reveals Metabolic Reprogramming in a Pair of Source-to-Sink Organs During Early Fruit Development in Tomato by LED Light Treatments

**DOI:** 10.3389/fpls.2018.01439

**Published:** 2018-10-09

**Authors:** Atsushi Fukushima, Shoko Hikosaka, Makoto Kobayashi, Tomoko Nishizawa, Kazuki Saito, Eiji Goto, Miyako Kusano

**Affiliations:** ^1^RIKEN Center for Sustainable Resource Science, Yokohama, Japan; ^2^Graduate School of Horticulture, Chiba University, Chiba, Japan; ^3^Graduate School of Pharmaceutical Sciences, Chiba University, Chiba, Japan; ^4^Graduate School of Life and Environmental Sciences, University of Tsukuba, Tsukuba, Japan

**Keywords:** *Solanum lycopersicum*, light emitting diode, metabolite profiling, RNA sequencing, light stress, fruit development

## Abstract

Tomato (*Solanum lycopersicum*) is a model crop for studying development regulation and ripening in flesh fruits and vegetables. Supplementary light to maintain the optimal light environment can lead to the stable growth of tomatoes in greenhouses and areas without sufficient daily light integral. Technological advances in genome-wide molecular phenotyping have dramatically enhanced our understanding of metabolic shifts in the plant metabolism across tomato fruit development. However, comprehensive metabolic and transcriptional behaviors along the developmental process under supplementary light provided by light-emitting diodes (LEDs) remain to be fully elucidated. We present integrative omic approaches to identify the impact on the metabolism of a single tomato plant leaf exposed to monochromatic red LEDs of different intensities during the fruit development stage. Our special light delivery system, the “simplified source-sink model,” involves the exposure of a single leaf below the second truss to red LED light of different intensities. We evaluated fruit-size- and fruit-shape variations elicited by different light intensities. Our findings suggest that more than high-light treatment (500 μmol m^-2^ s^-1^) with the red LED light is required to accelerate fruit growth for 2 weeks after anthesis. To investigate transcriptomic and metabolomic changes in leaf- and fruit samples we used microarray-, RNA sequencing-, and gas chromatography-mass spectrometry techniques. We found that metabolic shifts in the carbohydrate metabolism and in several key pathways contributed to fruit development, including ripening and cell-wall modification. Our findings suggest that the proposed workflow aids in the identification of key metabolites in the central metabolism that respond to monochromatic red-LED treatment and contribute to increase the fruit size of tomato plants. This study expands our understanding of systems-level responses mediated by low-, appropriate-, and high levels of red light irradiation in the fruit growth of tomato plants.

## Introduction

Tomato (*Solanum lycopersicum*), a member of the Solanaceae family, is the leading vegetable crop. Supplementary lighting [e.g., fluorescent- and high-pressure sodium lamps, and light-emitting diodes (LEDs)] is used for tomato production in Northern Europe and Canada (for example, see Heuvelink et al., 2006). It can compensate for low rates of photosynthesis and increases both the growth and yield of tomato plants when compared to natural light (Gosselin et al., 1996; Gunnlaugsson and Adalsteinsson, 2006). Most greenhouses and areas without sufficient daily light integral (DLI) require such supplementary lights to maintain the optimal light environment for the stable growth of tomato plants. A seasonal effect of supplementary light was observed throughout the year (except from June to August); it resulted in increases in the tomato yield (Heuvelink et al., 2006). Others documented that supplementary lighting had no- or negative effects (Gunnlaugsson and Adalsteinsson, 2006; Trouwborst et al., 2010). These observations suggest that DLI from natural and supplemental lighting per plant, the light source, and/or the cultivar play an important role in determining fruit growth rates and yield. Also, depending on the crop species and several growth factors (e.g., temperature, CO_2_, and air humidity), the light intensity [photosynthetic photon flux (PPF in μmol m^-2^ s^-1^)] should be optimized to provide sufficient supplementary lighting without eliciting leaf stress and associated leaf disorders (Moe et al., 2006; Darko et al., 2014).

Several tomato fruit characteristics, mainly the result of dramatic metabolic shifts during development and ripening, result in a complex system (Carrari and Fernie, 2006; Bovy et al., 2007). Increasing the fruit yield per plant is important but challenging as the molecular mechanism of the source-to-sink balance, a key step toward fruit development, remains largely unclear. The translocation of carbohydrates like sucrose and other nutrients from source to sink is a major determinant of plant growth (Nguyen-Quoc and Foyer, 2001; Paul et al., 2001; Ruan, 2014). Plants strictly regulate the production of photoassimilates and the source-to-sink response to changing environments (Lemoine et al., 2013; Osorio et al., 2014). Of these, sucrose contributes to translocation as a main carbon source in phloem. Tomato plants overexpressing sucrose phosphate synthase (SPS), a key enzyme in the sucrose metabolism, exhibited substantially altered carbon allocation in photosynthetic leaves (Galtier et al., 1993, 1995; Micallef et al., 1995). A reduction in the activity of sucrose synthase (SuSy), which catalyzes the sucrose cleavage in tomato fruit, considerably reduced its sucrose unloading capacity (D’Aoust et al., 1999). A comprehensive and quantitative molecular understanding of the tightly coupled coordination of photosynthesis and sink capacity is important. With respect to the quality of tomato fruit, these systems are closely associated with the phloem loading of sucrose in the source and with unloading in sink tissues via the central carbon metabolism, although generally, photosynthesis in fruit is not essential (Kahlau and Bock, 2008; Lytovchenko et al., 2011).

Transcriptome analysis with microarrays and RNA-sequencing (RNA-Seq) revealed important key factors involved in fruit ripening (Lin et al., 2008; Chung et al., 2010; Nakano et al., 2012; Fujisawa et al., 2014; Nguyen et al., 2014). The integration of transcriptomic and metabolomic approaches demonstrated that the detected primary metabolites, cell wall-related metabolites, and pigments were not strongly correlated with known key genes involved in ripening, but implied a causal relationship between tricarboxylic acid (TCA) cycle intermediates and fruit ripening (Alba et al., 2005; Carrari et al., 2006; Mounet et al., 2009; Osorio et al., 2011). Despite the agricultural importance of the developmental process under supplementary lighting, the comprehensive metabolic and transcriptional behaviors along the developmental period remain to be fully elucidated. Also, their role under artificial supplementary lighting with LEDs (Goto, 2003; Darko et al., 2014) in the regulation of flowering and early fruit development (rather than fruit ripening) remains to be identified quantitatively and systematically.

We present integrative omics approaches to elucidate the metabolomic impact of red LED light of different intensities on single leaves during the early fruit development of tomato plants. We set up a special light-irradiation system, our “simplified source-sink model,” which involves a single tomato leaf, a fruit truss, and monochromatic red-LED light delivered during early fruit development; red light is widely used for supplemental lighting. Our findings suggest that the proposed workflow promises to aid in the discovery of key pathways that contribute to increasing the fruit size of tomatoes.

## Materials and Methods

### Plant Material and Growth Conditions

Seeds from tomato (*Solanum lycopersicum* ‘Reiyo’) were sown in 72-cell trays (Takii Seed, Kyoto, Japan) and grown in a soil mix (Napura Soil Mixes, Yanmar, Osaka, Japan) for 2 weeks in a growth chamber (MKV DREAM, Tokyo, Japan) at 25°C/20°C (light/dark, Japan) and 900 μL L^-1^ CO_2_ concentration. The light/dark cycle was 16 h/8 h for 2 weeks. Then the seedlings were transferred to 2.4 l pots and grown in a growth chamber (Asahi Kogyosha, Tokyo, Japan); the PPF level was adjusted to 450–500 μmol m^-2^ s^-1^ when measurements were at the meristem of each tomato plant (light source: ceramic metal halide lamps). *S. lycopersicum* cv. ‘Moneymaker’ was also used and exposed to the same conditions of cv. ‘Reiyo’ for fruit measurements. The experiments were performed at Chiba University, Japan.

For LED irradiation, we exposed single leaves for 4 weeks to a red LED panel (23 cm × 12 cm, 18 W, Shibasaki, Saitama, Japan); the peak wavelength was 660 nm (Showa Denko K. K., Tokyo, Japan). To remove the effects of supplemental light from other factors, we removed all leaves and trusses except for the flowers on the second truss, the leaf just below the second truss, and the apical portions of the main shoot at the anthesis stage of the second truss (Hikosaka et al., 2013) (**Supplementary Figure S1**). Each plant was trimmed to bear a single leaf and a truss with three flowers (**Supplementary Figure S1B**). We used four light intensities at PPF 0-, 200-, 500-, and 1,000 μmol m^-2^ s^-1^ (P0, P200, P500, P1000). Different PPFs were applied to post-anthesis tomato plants for 2 weeks after anthesis (WAA), corresponding to 14 days after anthesis (DAA). Leaf and fruit samples were harvested 0-, 1-, and 2 WAA at the same hour 1600 (JST, Japan Standard Time), corresponding to midday in the growth chamber.

### Fruit Measurements

It is known that the fresh weight (FW, in g) of tomato fruit can be estimated from the fruit length, diameter, and height (for example, see Mutschler et al., 1986). Therefore, fruit measurements were taken every week with a digital caliper and recorded as the estimated FW of each fruit. Biological replicates, *n* = 3.

### RNA Isolation

Total RNA was isolated using the RNeasy Plant Mini kit (Qiagen, Hilden, Germany) according to the manufacturer’s instructions. The concentration, integrity, and extent of contamination by ribosomal RNA were monitored using an ND-1000 spectrophotometer (Thermo Fisher Scientific, Waltham, MA, United States) and a Bioanalyzer 2100 (Agilent Technologies, Santa Clara, CA, United States).

### cDNA Library Construction and RNA-Sequencing

Beads with oligo(dT) were used to isolate poly(A) mRNA after total RNA was collected from tomato tissues, leaves, and fruits. Fragmentation buffer was added to cut mRNA into short fragments to serve as templates; random hexamer primer was used to synthesize first-strand cDNA. Second-strand cDNA was synthesized using buffer, dNTPs, RNaseH, and DNA polymerase I. Short fragments were purified with the QiaQuick PCR extraction kit and resolved with EB buffer for end repair and for adding poly(A). The short fragments were connected with sequencing adapters. After agarose gel electrophoresis, suitable fragments were selected as templates for polymerase chain reaction (PCR) amplification. Lastly, the library was sequenced using Illumina HiSeq^TM^ 2000. We complementary analyzed same samples used for microarray analysis per condition (*n* = 1 each).

### Sequence Processing, Mapping Reads to a Reference, and Differential Expressions

After Illumina reads were quality-checked, demultiplexed and trimmed, they were clustered per library using RobiNA (Lohse et al., 2012). The remaining short reads used for assembly were aligned to the CDS sequences with Bowtie (Langmead et al., 2009) to identify rRNA contamination; two mismatches were allowed. The ribosomal filtered reads were then aligned against tomato genome sequence SL2.40 (ITAG2.3) (Tomato Genome, 2012). Differentially expressed genes (DEGs) were identified using the DESeq (Anders and Huber, 2010) with default parameters. The level of significance was set at a false discovery rate (FDR) < 0.05 (Benjamini and Hochberg, 1995). We used BiNGO (Maere et al., 2005) to analyze significantly over-represented gene ontology (GO) categories in the DEGs (FDR < 0.05).

### Metabolite Profiling

Metabolite profiling by gas chromatography-time-of-flight mass spectrometry (GC-TOF-MS) was performed essentially as described (Kusano et al., 2007a,b, 2011a) but with tomato-specific modifications [see our meta-data (accession no. MTBLS699) in MetaboLights (Kale et al., 2016)]. Briefly, all raw data in netCDF format were pre-processed by hyphenated data analysis (HDA) (Jonsson et al., 2005, 2006) and the obtained data matrix was normalized and summarized using the cross-contribution compensating multiple standard normalization (CCMN) method (Redestig et al., 2009). For metabolite identification, we cross-referenced the obtained mass spectra with gas chromatography with electron impact mass spectrometry (GC-EI-MS) and retention index libraries (Schauer et al., 2005) in the Golm Metabolome database (Kopka et al., 2005) and our own in-house libraries. According to the recommendation (Fernie et al., 2011), detailed information on metabolite identification was shown in **Supplementary Tables S2A,B**. The metabolite profile data (processed data) with our experimental design (phenodata) are also included in **Supplementary Tables S2G,H**. We compared the metabolite responses: (1) treatment comparison, i.e., highlight vs. lowlight treatment and (2) developmental comparison, e.g., 2 WAA vs. 1 WAA under LED irradiation at P1000. The control condition of comparison (1) was P200 red light, whereas 1 WAA was used as control condition in the case of (2). Each sample point was analyzed with six biological replicates.

### Statistical Data Analyses for Transcript Profiling by Microarrays and Metabolite Profiling

We used same microarray data that were analyzed in our previous study (Fukushima et al., 2012) [accession#: GSE35020 in NCBI GEO (Barrett et al., 2013)]. We re-analyzed the total of 18 samples (12 leaf- and 6 fruit samples); three biological replicates per sample were used. Data normalization, visualization, and correlation analysis based on Pearson’s correlation were performed using R^1^ and Bioconductor (Gentleman et al., 2004). DEGs and differentially accumulated metabolites were identified using the LIMMA method, which is based on linear model fitting (Smyth, 2004). The level of significance was set at FDR < 0.05 (Benjamini and Hochberg, 1995). Principal component analysis (PCA) was performed using the pcaMethods package (Stacklies et al., 2007), with log-transformation and unit variance scaling. To visualize the global transcript responses of gene regulatory networks and metabolic pathways of fruit- and leaf samples, we used MapMan software (v3.5.1R2) (Usadel et al., 2005). Genes were classified into different functional categories based on MapMan BIN from the ITAG2.4 annotation. We used BiNGO (Maere et al., 2005) to analyze significantly over-represented GO terms in the DEGs. The level of significance was set at FDR < 0.05 (Benjamini and Hochberg, 1995).

## Results

### Scope of the Study and Its Systematic Experimental Design

In preliminary experiments, we studied the fruit weight and leaf area of whole *Solanum lycopersicum* L., ‘Reiyo’ plants exposed or not exposed to red LED irradiation. We first recorded the fruit weight along the developmental stages 1-, 2-, 3-, and 4 WAA of plants grown without supplemental LED lighting (**Supplementary Figure S2A**). Under normal light (average P500, metal halide lamp without supplemental LED light), we observed a remarkable increase in the fruit weight between 1- and 2 WAA, suggesting that the period was critical for early fruit development and the time of cell expansion. When we recorded the fruit weight and leaf area of whole plants grown under supplemental red LED light (P1000), we detected no effect on the fruit biomass at 2 WAA (**Supplementary Figure S2B**).

Based on these preliminary findings we focused on 2 WAA and developed a custom LED light system to gain insights into molecular regulation governing early tomato fruit development and biologically relevant changes in the storage pattern and translocation under different light conditions. Our “simplified source-sink model” (Hikosaka et al., 2013) is comprised of a single tomato leaf and fruit truss (**Supplementary Figure S1**) and can be used to deliver red or other color light irradiation in greenhouses or under closed growth conditions, e.g., in climate chambers. Although we delivered 100% of red LED light to the plants, leaves exhibited few stress signs under the P200 condition. However, after P500 and P1000 high light (HL) exposure, they manifested stress signs and accompanying disorders, including leaf curling and senescence (**Supplementary Figure S3**), due mainly to high light intensity and seemingly enhancement of translocation.

### Enhanced Light Intensity Strongly Affects Leaf and Fruit Growth in Tomato

**FIGURE 1:**
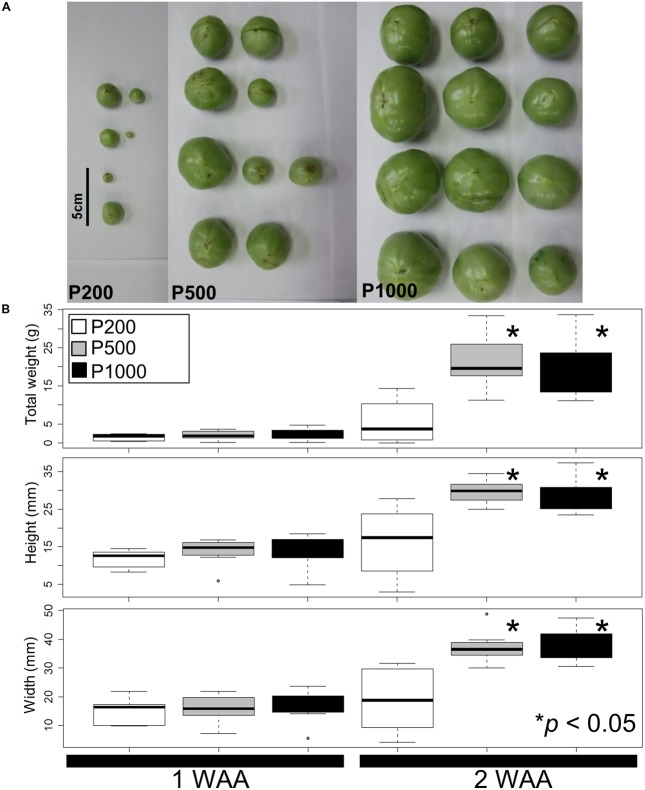
Fruit size and shape variations elicited by different light intensities. **(A)** A representative fruit shape developed under red LED panels (P200, P500, and P1000) in 2 WAA. Scale bar: 5 cm. **(B)** Measured tomato fruit sizes. Statistically significant differences between fruits exposed to light intensities at P200, P500, and P1000. We used a box and whisker plot, a graphical summary of a distribution. This plot can visualize the minimum, lower and upper quartiles (25% and 75%), median, and maximum of data. Regarding extreme values, outliers may be displayed as open circles. Data show the mean of the total weight, height, and width calculated with the Welch *t*-test. Differences of ^∗^*p* < 0.05 were considered statistically significant. The samples were used for metabolite profiling. The results indicate that treatment with higher than P500 red LEDs is sufficient for fruit growth under our artificial conditions. Biological replicates, *n* = 3. WAA, week after anthesis.

Using our simplified source-sink model we next assessed variations in the fruit size and shape due to different light intensities. **Figure 1A** shows a representative fruit shape developed under red LED irradiation (P200, P500, and P1000) in 2 WAA. Although the tomato plants were grown simultaneously in a controlled growth chamber under artificial conditions, there were variations in the fruit morphology due to uncontrollable factors affecting fruit set (**Supplementary Table S1**). For example, the developmental stage at P1000 irradiation in 4 WAA can correspond to breaker (in some case, it corresponds to red ripe). In the case of P500 in 4 WAA, the stage corresponds to mature green. The mean weight, height, and width were statistically greater after HL- than P200 treatment (*p* < 0.05, Welch’s *t*-test) (**Figure 1B**). We also evaluated variations in the fruit size and shape obtained under the same conditions in a different year (i.e., independent *S. lycopersicum* L. ‘Reiyo’ experiments) (**Supplementary Figure S4**). *S. lycopersicum* cv., ‘Moneymaker,’ exposed to the same conditions also exhibited this tendency (**Supplementary Figure S5**). Our findings suggest that treatment with red LED light exceeding P500 is sufficient for fruit growth in tomato plants grown under our artificial conditions.

### Overview of Metabolite and Transcript Responses to High Light Irradiation

**FIGURE 2:**
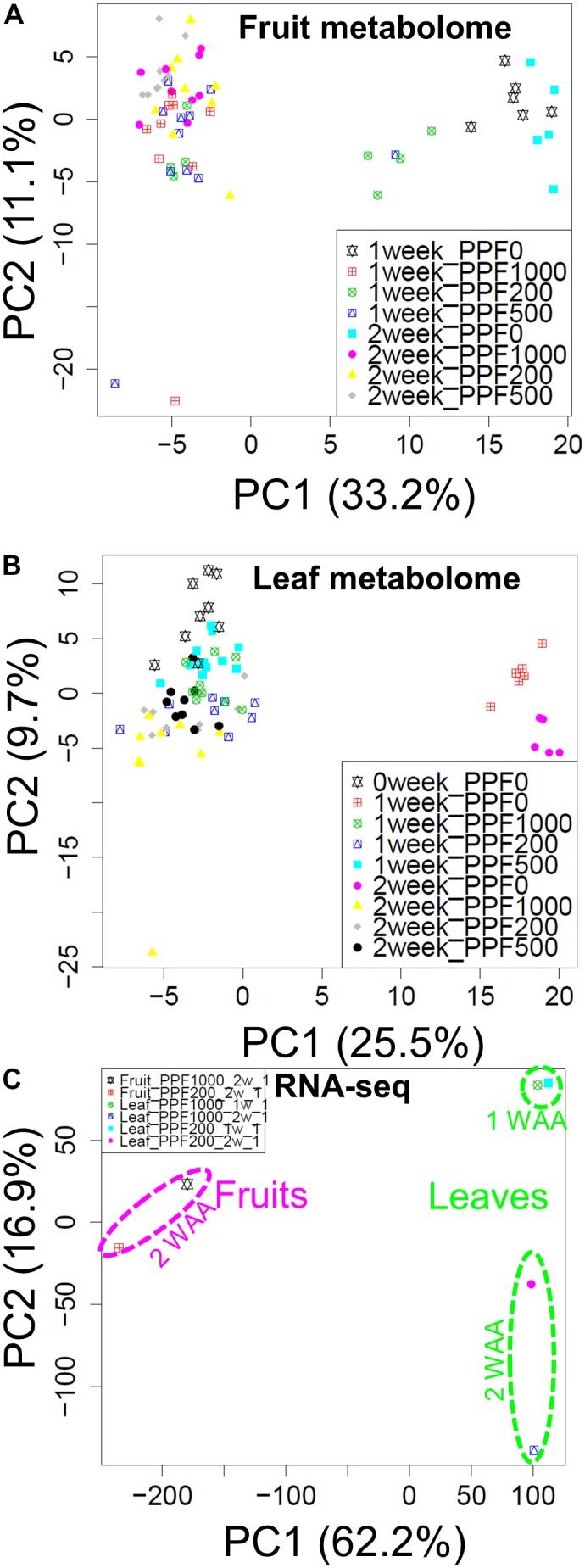
Overview of the transcript and metabolite responses. Score scatter plots of the fruit metabolome **(A)**, and the leaf metabolome **(B)** obtained by GC-TOF-MS, and of the transcriptome obtained by Illumina-based RNA-Seq **(C)**. We observed changes in the metabolite composition of fruits and leaves in the presence or absence of light (PC1). RNA-Seq data also revealed different cluster groups according to tissue-dependent differences in the PC1 axis. The recorded changes in the growth stages were observed under PC2. Biological replicates, *n* = 5–6 for metabolite profiling and *n* = 1 for transcript profiling obtained by RNA-Seq analysis.

To study small-molecule metabolites and gene expressions during early fruit development under red LED light with different light intensities, we performed global metabolite and transcript profiling using the experimental designs of an established GC-MS method (Kusano et al., 2007a,b), Illumina-based RNA-Seq, and previously reported microarrays (Fukushima et al., 2012) (**Supplementary Figures S6**–**S8**). To visualize the extent of metabolomic and transcriptomic changes elicited by different light intensities, we performed PCA and applied the data matrices of the metabolite- and transcript profiles separately. The PCA score scatter plot revealed that the strong impact on metabolite levels in accordance with observance of the presence or absence of light along with PC1 (**Figures 2A,B**). RNA-Seq data (**Supplementary Figure S9**) also showed different clustering groups based on tissue-dependent differences in the PC1 axis, while growth stages with PC2 (**Figure 2C**). When we focused on light intensity-dependent metabolomic changes in fruits, we found that samples exposed to HL conditions were clustered, while samples with light treatments were clearly separated from dark samples (P0). These observations suggest that while our HL condition strongly affected the metabolite accumulation in tomato plants, its effect on developing fruit was not as large.

### Comparative Analyses of Metabolite Profiling of Tomato Fruit- and Leaf Samples Under Different Light Intensities

**FIGURE 3:**
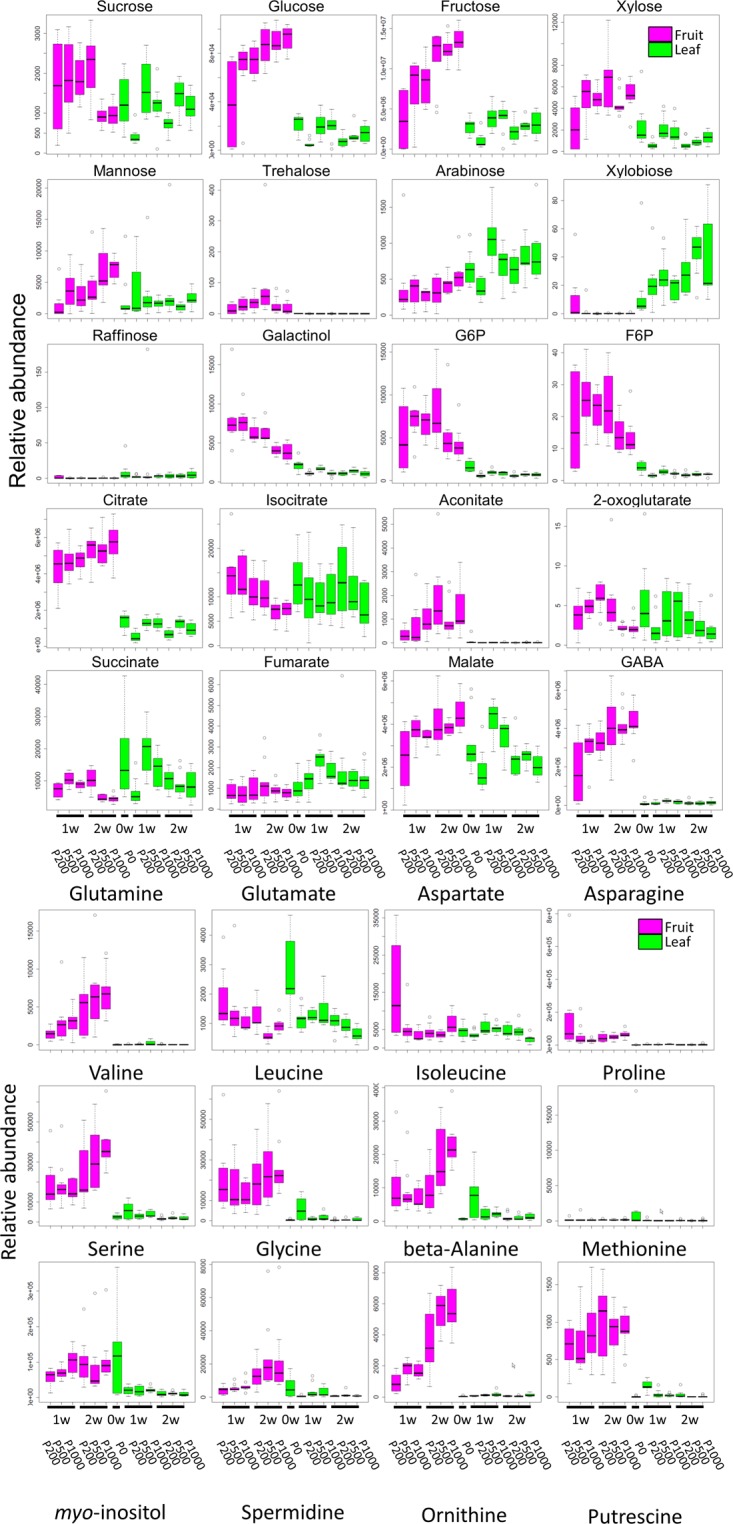
Comparative analyses of metabolite profiling of fruit- and leaf samples of tomato plants grown under different light intensities (P0, P200, P500, and P1000). To summarize metabolite profile data, we used a box and whisker plot, a graphical summary of a distribution. This plot can visualize the minimum, lower and upper quartiles (25% and 75%), median, and maximum of each metabolite data. Regarding extreme values, outliers may be displayed as open circles. In each boxplot, the investigated time-points are 1 WAA and 2 WAA (*X*-axis). The relative abundance on the *Y*-axis shows the normalized responses of the metabolite peaks obtained by GC-TOF-MS. Typical metabolite classes such as sugars, sugar alcohols, and amino- and organic acids are shown. Magenta- and green boxes represent samples from fruit and leaves, respectively. Biological replicates, *n* = 5–6. WAA, week after anthesis.

Our broad-range metabolite analysis identified HL-responsive metabolites and revealed changes in metabolite levels throughout the early fruit-development stage (**Figure 3**). We first focused on metabolites that exhibited a statistically significant difference when the plants were grown under HL and under control conditions (**Table 1** and **Supplementary Table S2**). When we compared between P1000 and P200 light intensities, we found that at P1000, sugars including glucose, fructose, and trehalose and cell wall related metabolites like xylose and mannose markedly increased in 1 WAA fruits while aromatic amino acids such as phenylalanine, tryptophan, and tyrosine, were decreased. There were fewer HL-responsive metabolites in 2 WAA than 1 WAA fruits, resulting in 10 significantly changed metabolites. Inverse changes were observed in the metabolite levels, for example, phenylalanine and tyrosine were increased in response to HL.

**Table 1:** Metabolite responses of tomato fruits to high light treatment.

	Log_2_FC	FDR
**Fruit 1 WAA, P1000/P200**		
Trehalose	4.8	1.10E-02
Mannose	4.3	2.90E-03
Fructose	3.8	8.70E-05
Glucose	2.5	2.30E-05
Xylose	2.2	3.10E-04
Butyro-1,4-lactam	2.1	9.70E-04
GABA	2	1.70E-03
Aconitate	1.9	2.70E-02
Dihydrouracil	1.5	7.90E-03
Threonate	1.4	2.90E-02
Aspartate	-1.7	7.50E-05
Lysine	-1.9	3.90E-02
Asparagine	-2.2	5.90E-04
Tryptophan	-2.4	4.60E-03
Alpha-tocopherol	-2.7	1.60E-02
Phenylalanine	-2.8	4.70E-04
Tyrosine	-3	2.70E-02
Xylobiose	-5.9	9.60E-04
**Fruit 2 WAA, P1000/P200**		
Tyrosine	3.3	2.50E-02
Phenylalanine	1.9	3.00E-02
Isoleucine	1.5	4.10E-02
Arabinose	1.4	3.00E-02
Threonine	1.1	4.10E-02
Succinate	-1.2	9.00E-03
Shikimate	-1.6	9.00E-03
Threonate	-2	6.20E-03
Dihydrouracil	-2.1	4.10E-04
Galacturonate	-6.3	2.00E-02

*The listed metabolites exhibited a statistically significant difference under HL and control conditions at 1- and 2 WAA [LIMMA (Smyth, 2004); |log_2_FC|≥ 1, false discovery rate (FDR) < 0.05]. Log_2_FC means logarithmically transformed values of fold-changes (e.g., P1000/P200). See also **Supplementary Table S1**. HL, high light; FC, fold-change.*

Except at 2 WAA, the trend observed in P500 and P1000 fruits was similar. Fruits examined in 2 WAA did not show a significant increase in metabolites; mainly polyol and some hydroxyl acids were decreased. Sucrose, glucose, and xylose increased in response to HL. The significantly increased organic acids in 1- and 2 WAA leaves exposed to HL were citrate, malate, succinate, quinate, and glycerate. Methionine levels in young leaves dramatically decreased in response to HL. In 1 WAA leaves, P500 HL largely affected the metabolome; the number of significantly accumulated metabolites was higher than at P1000 HL although we observed a similar tendency at P500 and P1000.

Comparison of 1- and 2 WAA fruits showed that sucrose decreased under HL while glucose and fructose increased in an essentially linear manner during fruit development and in response to HL (**Figure 3** and **Supplementary Table S2E**). Cell-wall related metabolites like xylose, mannose, and arabinose increased during fruit development. Irrespective of the light intensity, the level of most sugars, sugar phosphates, and some organic acids like citrate and aconitate was higher in fruits than leaves (**Supplementary Table S2F**). Most highly accumulated amino acids in fruits were γ-amino butyrate (GABA), glutamine, asparagine, branched chain amino acids (valine, leucine, and isoleucine), beta-alanine, and methionine.

### Genome-Wide Transcript Profiling Revealed a Wide Range of Variations in Gene Expression and Reflected Changes in Regulatory Networks Under High Light Treatment

**FIGURE 4:**
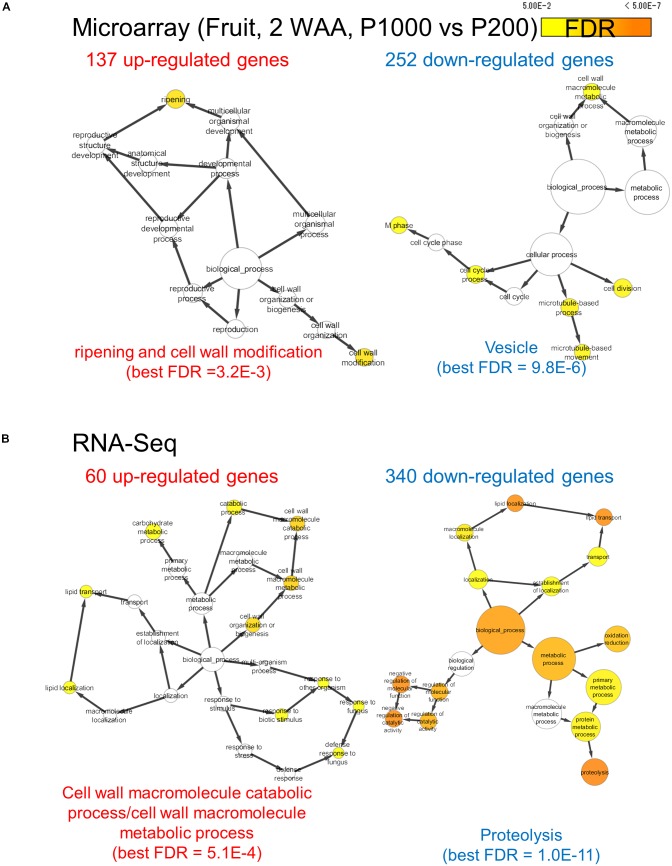
Gene ontology term enrichment analysis for HL-responsive genes in tomato fruits (2 WAA, P1000 vs. P200). We used microarray **(A)** and RNA-Seq data **(B)**. BH FDR < 0.05, |log_2_FC|≥ 1. WAA, week after anthesis.

We assessed the comprehensive transcript abundance using microarrays and RNA-Seq (**Supplementary Tables S3**, **S4**). To mitigate issues associated with the coverage of gene annotation in microarrays we also performed RNA-Seq with the Illumina-based platform (**Supplementary Figure S9**). Using both datasets we identified DEGs. At P1000 and P200, microarray-based approaches detected 137 up- and 252 down-regulated genes, respectively, in fruits (**Figure 4A** and **Supplementary Table S5**). GO enrichment analysis in “Biological Process” showed that the 137 up-regulated genes were significantly enriched in “ripening (FDR = 3.2E-3)” and “cell wall modification (FDR = 3.2E-3),” whereas the 252 down-regulated genes were related to, for example, “cell division (FDR = 1.8E-2)” and “microtubule-based process (FDR = 1.4E-2).” Ripening-related genes encoded pectin methylesterase PME2.1 (probeset ID = Les.3630.1.S1_at), expansin 1 (probeset ID = Les.191.1.S1_at), and PME1.9 (3 probeset IDs: Les.3122.1.S1_a_at, Les.3122.2.A1_at, and Les.3122.2.A1_a_at). The RNA-Seq-based approach identified 60 up- and 340 down-regulated genes between P1000 and P200 treatments in tomato fruit (**Figure 4B** and **Supplementary Table S5B**). GO enrichment analysis using DEGs obtained by RNA-Seq indicated that the 60 up-regulated genes were involved in the “cell wall macromolecule catabolic and metabolic process (FDR = 5.1E-4)” and in lipid localization/transport (FDR = 0.047) (**Figure 4B**). The 340 down-regulates genes were related to “proteolysis (FDR = 1.1E-11)” and “negative regulation of molecular function (FDR = 9.3E-7).”

RNA-Seq of 1 WAA leaves also revealed that DEGs that were up-regulated at P1000 and were related to biological processes like “regulation of transcription (FDR = 1.2E-4)” included genes that encode MYB-related transcription factor, WRKY-like MYB-related transcription factor, and heat-shock factor protein. Our microarray data supported this observation (**Supplementary Table S5**). The number of down-regulated DEGs was larger than of up-regulated DEGs in P1000 2 WAA leaves; we only observed eight up-regulated DEGs. Our analysis for down-regulated genes significantly over-represented “glycerol metabolic process (FDR = 7.2E-4),” “alditol metabolic process (FDR = 7.2E-4),” and “polyol metabolic process (FDR = 1.3E-2).” Together, the results of our global transcript analysis suggest the presence of highly complex transcription dynamics in tomato fruits and leaves exposed to P1000 and P200 and examined at different developmental stages. These findings are reflected as the systems-level response to HL under artificial condition using red LEDs.

### Tomato Leaf and Fruit Samples Exhibited Inverse Changes in the Expression Patterns Involved in Light Reactions, Secondary Metabolism, and Cell-Wall Biosynthesis

**FIGURE 5:**
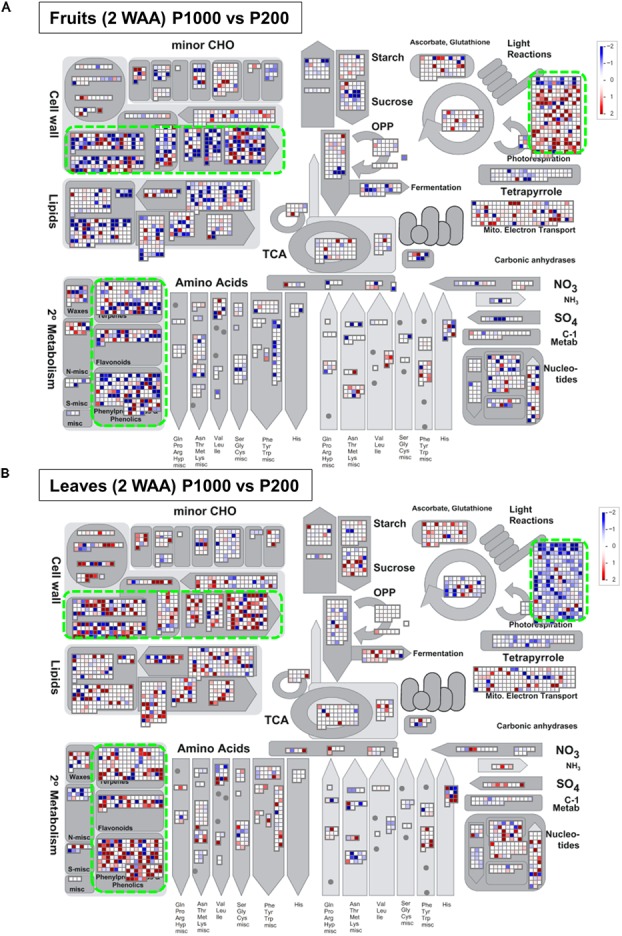
Overview of the transcript profiles based on Illumina-based RNA-Seq. The RNA-Seq data from **(A)** fruit- and **(B)** leaf samples were obtained with MAPMAN software (http://mapman.gabipd.org/web/guest/mapman) (Thimm et al., 2004; Usadel et al., 2005). The fold-changes are presented in different colors where red = up-regulated and blue = down-regulated by P1000 treatment. The results show that the changes in the expression patterns involved in light reactions, the secondary metabolism, and in cell-wall biosynthesis were inverse in fruit- and leaf samples (green rectangles). WAA, weeks after anthesis.

For a comprehensive study of transcript level changes in metabolic pathways, we visualized our RNA-Seq data using MAPMAN (Thimm et al., 2004; Usadel et al., 2005). **Figure 5** presents an overview of the transcript profiles of 2 WAA fruit- and leaf samples exposed to P1000- or P200 light treatment (**Supplementary Figure S10**). MapMan analysis demonstrated that, as a whole, changes in the expression patterns involved in light reactions, the secondary metabolism, and cell-wall biosynthesis exhibited an inverse tendency in fruit- and leaf samples. The marked up-regulation in the transcript level of P1000 fruits was associated with light reactions; in leaves those genes were down-regulated in response to HL. In leaves, genes involved in cell-wall biosynthesis and secondary metabolism were up-regulated, in fruits they were down-regulated.

**FIGURE 6:**
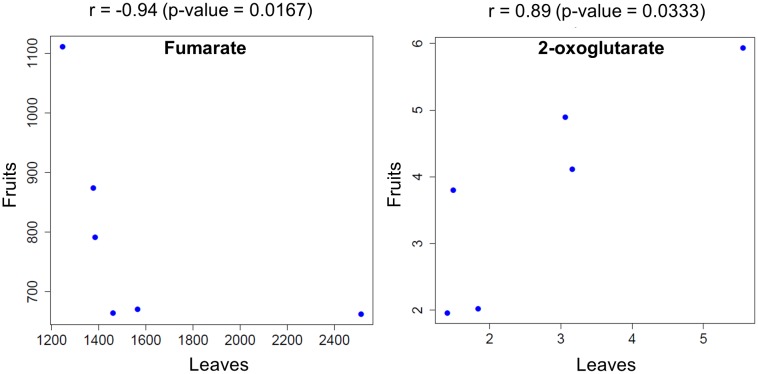
Metabolites that were significantly correlated between fruit- and leaf samples across the examined time points. Circles represent the mean level of metabolites obtained at each time point for plants exposed to different light intensities.

To shift our focus onto dissecting the metabolic balance in fruit- and leaf samples as a whole plant system, we used Spearman’s correlation (*p* < 0.05) to identify metabolites that exhibited a significant correlation in fruit and leaf samples across time. We found that fumarate showed a negative correlation between fruit and leaf samples, while 2-oxoglutarate exhibited a positive correlation (**Figure 6**).

## Discussion

To enhance the growth/yield and to improve the fruit quality of tomato plants, a physiological understanding of their metabolic and transcriptional responses during fruit development under artificial supplementary LED light is necessary. In our “simplified source-sink model” (Hikosaka et al., 2013) (**Supplementary Figure S1**), each tomato plant is pruned to have a single leaf and one fruit truss. To gain insights into the storage patterns and translocation in developing tomato fruits in response to environmental perturbation by HL irradiation (**Figure 1**), we performed sophisticated molecular phenotyping of samples exposed to red-LED lighting (660 nm peak). Metabolite and transcript analysis using GC-MS and RNA-Seq/microarrays enhanced our understanding of the cellular response of the fruit storage metabolism associated with primed fruit ripening and cell-wall biosynthesis, the stress response, and photosynthesis in response to the light wavelength and to HL stress imposed by our artificial irradiation system (**Figure 2**). Our findings emphasize the tightly coupled coordination of photosynthesis and sink capacity and provide an important list of the candidate metabolites, transcripts, and key pathways that contribute to the cellular metabolic shift in the course of early fruit development.

Our “simplified source-sink model” is appropriable because it removes as many unwanted variations due to unstable greenhouse conditions as possible. We used this experimental system in our earlier co-expression network analysis to infer candidate functional genes in tomato plants (Fukushima et al., 2012). Applying the experimental system also enabled to compare the extent of changes of light intensity, the photosynthesis rate, and fruit growth in tomato plants grown under two types of supplementary LED lighting methods (Hikosaka et al., 2013). First, they assessed the effects of LED light intensity on the fruit set, dry weight, dry mass ratio of a tomato fruit, and the net photosynthetic rate of a center leaflet, i.e., by applying the same method presented in the study. The second experiments in the study assessed whether number of leaves irradiated by supplemental lighting made effects on the photosynthetic rate of a whole tomato plant. As the light irradiation per leaf could increase photosynthetic rate in both experiments, the present study performed comprehensive molecular phenotyping of samples collected under different light intensities by applying the simplified experimental design.

An artificial environment can cause plant developmental and morphological differences, and their responses can mask essential traits. The plant response to a combination of multiple abiotic stresses in the field condition cannot be directly extrapolated from that to each stress exposed individually (Mittler, 2006; Mishra et al., 2012). In addition, we notice an emerging area, so-called ‘Field Omics’ (Alexandersson et al., 2014), trying to monitor and analyze different molecular behavior of samples harvested from crop field trials. The current field-omics approaches face a difficult and fundamental problem causing from high variance influenced by temporal and spatial differences in field trials. Such intrinsic heterogeneity in each field overwhelms effect of the experimental perturbations. At least partially, our data can be used as one of references to learn about the differences in molecular mechanisms between field- and laboratory tests, although our system greatly differs from the field trials.

To increase and improve light distribution, supplementary lighting is widely used as it can promote a good photosynthetic response and fruit growth in the lower plant layers. Others (Gosselin et al., 1996; Hovi et al., 2004; Gunnlaugsson and Adalsteinsson, 2006; Hovi-Pekkanen and Tahvonen, 2008; Pettersen et al., 2010) reported the positive effects of supplemental lighting on fruit growth and yield. Based on our evaluation of the tomato fruit size and of shape variations, exposure to around P500 red LED light is sufficient for fruit growth not only with respect to *S. lycopersicum* L. ‘Reiyo’ but also *S. lycopersicum* cv. ‘Moneymaker’ (**Supplementary Figures S3**–**S5**). To confirm whether this observation is cultivar-dependent or independent, a larger number of samples and cultivars should be needed in a future study. As we used only monochromatic red LED panel in this study, future studies will also focus on other light quality treatments (e.g., blue and mixture of red and blue).

Our study highlighted metabolic shifts in the carbohydrate metabolism and in several key pathways that may contribute to early fruit development under HL condition (**Figures 2**–**4**). A wide range of plant metabolic responses to various stresses has been studied by metabolomic- and transcriptomic approaches (Shulaev et al., 2008; Urano et al., 2010; Obata and Fernie, 2012; Nakabayashi and Saito, 2015; Noctor et al., 2015). While plants need light for photosynthesis and their healthy growth, it can damage plant cells; strong light irradiance is an abiotic stress factor. Genome-wide analyses can be used to characterize plant stress responses to high (excess) light stress and it can contribute to enhancing our understanding of stress-signaling pathways and the central metabolism including glycolysis, the TCA cycle, and photorespiratory pathways (Obata and Fernie, 2012). Besides identifying HL stress-responsive metabolites like sucrose, inositol, and GABA in tomato leaves, we found that HL stress (both P1000 and P500) led to dramatic decreases in aromatic amino acids in fruits at the early developmental stage (**Table 1** and **Supplementary Table S1**). We also showed that the expression patterns associated with light reactions, the secondary metabolism, and cell-wall biosynthesis exhibited inverse changes when we compared fruit- and leaf samples (**Figure 5**). The coordinated expressions associated with light reactions indicate functional photosynthesis in immature green fruit of tomato plants, which is consistent with early reports (Piechulla et al., 1987; Wanner and Gruissem, 1991; Schaffer and Petreikov, 1997; Alba et al., 2004; Kahlau and Bock, 2008; Lytovchenko et al., 2011). In fruits, down-regulated genes were involved in cell wall degradation. For example, there were down-regulated genes encoding a polygalacturonase and a pectate lyase. Both genes are known to be up-regulated and their activities become dominant during tomato fruit ripening (Cheng and Huber, 1997). In leaves, the coordinated expressions involved in phenylpropanoid- and flavonoid biosynthesis included up-regulated genes encoding chalcone synthase (CHS) and dihydroflavonol 4-reductase (DFR). This implies that the both early flavonoid biosynthetic pathway- and the more anthocyanin-specific genes response to mitigate high light stress (Wulff-Zottele et al., 2010; Caldana et al., 2011; Kusano et al., 2011b).

We observed positive correlation relationships of 2-oxoglutarate between fruit and leaf samples (**Figure 6**). Recent works based on metabolite flux analysis and metabolic network models demonstrated that metabolite provision via TCA cycle has been operated in response to demand of physiological status in the cell (Sweetlove et al., 2010). Among the intermediates in TCA cycle, 2-oxoglutarate is a key compound relating to carbon-nitrogen metabolism (Hodges, 2002; Foyer et al., 2011; Araujo et al., 2014). Antisense of 2-oxoglutarate dehydrogenase complex, involving in enzyme reaction of 2-oxoglutarate as a substrate, exhibited reduction of tomato fruit biomass (Araujo et al., 2012). After inhibiting 2-oxoglutarate dehydrogenase complex in potato tuber, there was the significant decrease in the level of 2-oxoglutarate, while fumarate level was unchanged (Araujo et al., 2008). These observations imply that at least 2-oxoglutalate level and biomass of reproductive/storage organs are likely to be positively coordinated in *Solanaceae* such as tomato and potato.

Attention has long been focused on striking shifts in cell-wall composition and pigments (Rose et al., 2004), the strict control of climacteric fruit ripening by phytohormones (Alba et al., 2005; Barry et al., 2005), source-sink regulation (Do et al., 2010), and the physiological transition during the parallel differentiation of photosynthetically active chloroplasts into chromoplasts, (for example, see Klee and Giovannoni, 2011; Pesaresi et al., 2014) that occur during development and ripening of tomato plants. Comprehensive molecular phenotyping using transcript and metabolite profiling showed that *Aux/IAA* and *ARF* genes play an important role in triggering the fruit set program (Wang et al., 2005; Rohrmann et al., 2011). Critical aspects of metabolic regulatory mechanisms, especially the central metabolism that controls fruit development in tomato plants, have been studied (Carrari et al., 2006; Osorio et al., 2011). Steinhauser et al. (2010) used a near-isogenic line population derived from a cross between *S. lycopersicum* ‘M82’ and *S. pennellii* to compare changes in the enzyme activity levels that can affect the plant metabolism during fruit development. The studies stressed that the plant metabolism and source-sink interaction can be strongly affected by genetic and environmental perturbations and their interactions.

Broad metabolite profiling that combines the use of multiple analytical platforms and our proposed system is required for assessing the plant secondary or specialized metabolism because, in response to artificial LED, changes in the protectants, antioxidants, and other pigments/nutrients like lycopene are largely unclear. A significant difference between our- and earlier studies is the use of LEDs to inspect and capture the precise responses to HL treatment of tomato leaves and fruits. Specific wavelengths and bandwidths generated by our red LEDs yield the specific red spectrum more efficiently than red filters combined with other light sources and elicit specific plant growth. The light spectrum strongly affects plant growth and development (Goto, 2003; Darko et al., 2014; Kitazaki et al., 2018) and the blue light spectrum near UV may increase the level of polyphenols such as anthocyanin as protectants and/or antioxidants (Seyoum et al., 2006). Massa (2008) suggested that certain light wavelengths may help to protect plants from attacks by insects and pathogens that elicit plant diseases.

**FIGURE 7:**
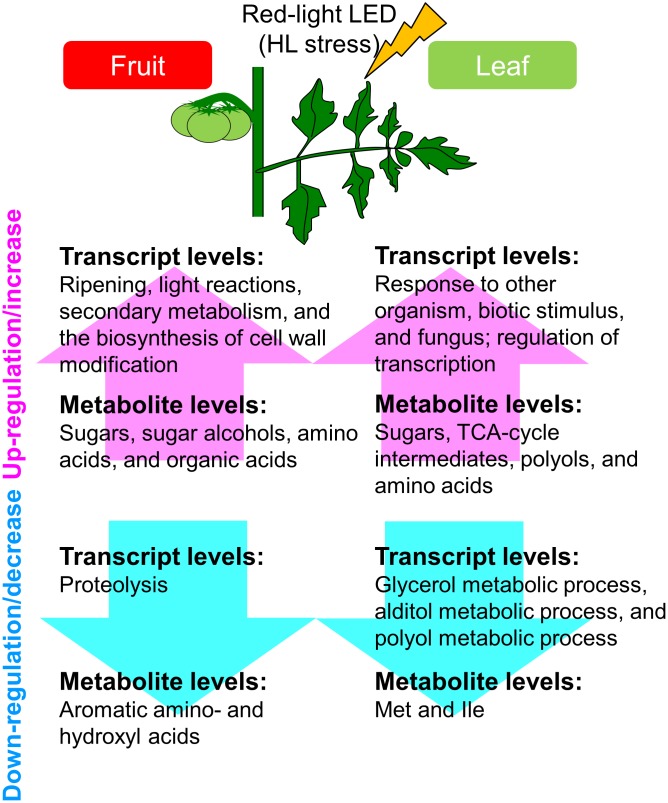
Schematic summary of the metabolic and transcriptional responses to high light treatment in ‘trimmed tomatoes’ grown under artificial light. Transcript profiling performed with microarrays and RNA-Seq showed that the expression patterns involved in light reactions, the secondary metabolism, and in cell-wall biosynthesis exhibited changes that tended to be inverse between fruit- and leaf samples. Metabolite profiling revealed that sugars and some organic acids, citrate, aconitate, and malate increased along the developmental stages in fruits. Amino acids like Gln, Val, Leu, Ile, and beta-alanine were abundant in fruits and tended to increase in the course of fruit development.

Finally, under strictly controlled systems and LEDs, tomato plants exhibited system-level dynamic behaviors in the metabolism (**Figure 7**). This was a precise plant response to the supplemental light source, i.e., red LED light, we used, and yielded new insights that differed from findings made when conventional filters were applied to broad-spectrum light. Our approaches avoid direct, unwanted large-scale effects resulting from unstable greenhouse conditions and different light intensities. Our strategy helps to deepen our understanding of systems-level responses during the growth of tomato fruit and provides fundamental resources for further studies to investigate the molecular basis of the high plasticity of the plant metabolism.

## Data Availability

Transcriptome datasets generated in this study are downloadable from the NCBI Sequence Read Archive (SRA) with the accession number DRA001843. Microarray GeneChip data are available at the NCBI GEO (GSE35020) as described in our previous study (Fukushima et al., 2012). All metabolite data (^∗^.netCDF format) are also downloadable from MetaboLights (Kale et al., 2016) (accession no. MTBLS699).

## Author Contributions

AF, SH, and MiK designed and performed the statistical data analyses and interpreted the data, and wrote the manuscript with contributions from the other co-authors. MiK, MaK, TN, and KS performed the transcript and metabolite profiling. SH and EG conducted and analyzed the measurement of physiological data. All authors read and approved the final manuscript.

## Conflict of Interest Statement

The authors declare that the research was conducted in the absence of any commercial or financial relationships that could be construed as a potential conflict of interest.

## Footnotes

1 http://www.r-project.org

## Supplementary Material

FIGURE S1 Simplified source-sink model and overall experimental design. **(A)** Schematic representation of red LED lighting in this study, named “simplified source-sink model.” We removed all the leaves and trusses except for the flowers of the second truss, the leaf just below the second truss, and the apical portions of the main shoot at the anthesis stage of the second truss. **(B)** Tomato plant cultivation with our systems under three types of light intensities (P200, P500, and P1000).

FIGURE S2 Fruit weight and leaf area under LED light irradiation in *Solanum lycopersicum* L., ‘Reiyo’ – a whole plant. **(A)** Fruit weight comparison along developmental stages under normal light condition (average PPF 500, metal halide lamp as ‘Control’) without supplemental LED lighting. Biological replicates, *n* = 3. **(B)** Measurement of fruit weight and leaf area under LED lighting (PPF 1000, red LED) at 14 days. A whole plant and a red LED panel were set in a semi-closed assimilation chamber. The leaf just below the second truss was irradiated by LED. The bar graph indicates mean and standard deviation. Biological replicates, *n* = 6. WAA, week after anthesis.

FIGURE S3 Tomato plants and leaf morphology on exposure to different light intensities of red LED at 2 WAA. **(A)** Leaf and fruit growth of tomato plants. **(B)** Leaf morphology. Few stress signs in the leaf were visible under P200 condition. The leaves indicated stress signs and accompanying disorders after P500 and P1000 high light treatments. Scale bars represent 10 cm.

FIGURE S4 Fruit size and shape variation under different light intensities in *Solanum lycopersicum* L., ‘Reiyo’ using “simplified source-sink model.” Measurement of fruit sizes **(A)** and leaf area **(B)**. WAA, week after anthesis. The bar graph indicates mean and standard deviation. Biological replicates, *n* = 1–6 for fruits and *n* = 3–6 for leaves.

FIGURE S5 Fruit size under different light intensities in *Solanum lycopersicum* L., ‘Moneymaker’ using “simplified source-sink model.” Measurement of fruit fresh weight **(A)** and dry weight **(B)**. Three fruits per plant/treatment. We used a box and whisker plot, a graphical summary of a distribution. This plot can visualize the minimum, lower and upper quartiles (25% and 75%), median, and maximum of data. Regarding extreme values, outliers may be displayed as open circles. Biological replicates, *n* = 3.

FIGURE S6 Experimental design for metabolite profiling. WAA, weeks after anthesis.

FIGURE S7 Experimental design for RNA-Seq analysis. WAA, weeks after anthesis.

FIGURE S8 Experimental design for microarray analysis. See in details, (Fukushima et al., 2012). WAA, weeks after anthesis.

FIGURE S9 **(A,B)** Statistics for sequenced short reads and mapping results of Illumina-based RNA-Seq. WAA, weeks after anthesis.

FIGURE S10 Overview of transcript profile based on Illumina-based RNA-Seq. The RNA-Seq data from leaf-samples (1 WAA) was described using MAPMAN software (http://mapman.gabipd.org/web/guest/mapman) (Thimm et al., 2004; Usadel et al., 2005). The fold change is visualized by pseudo-color: red, up-regulated by P1000 treatment; blue, down-regulated by P1000 treatment. The results show that expression patterns involved in light reactions, secondary metabolism, and the biosynthesis of cell wall exhibit opposite alteration tendency between fruit- and leaf samples (green rectangles). WAA, weeks after anthesis.

TABLE S1 Fruit developmental stages used for the study.

TABLE S2 Reporting metabolite data by GC-TOF-MS in this study. **(A)** Metabolite reporting checklist. **(B)** Summary of values for detected metabolites. This table is based on reporting suggestions (Fernie et al., 2011). Significantly changed metabolites, processed data matrix, and sample information are as follows. **(C)** Tomato fruits. Metabolite responses during the developmental stages. **(D)** Leaves. Metabolite responses to high light treatment. **(E)** Tomato fruits. Metabolite responses during the developmental stages. **(F)** Leaves. Metabolite responses during the developmental stages. **(G)** Summary of metabolome data matrix, and statistical data analysis. **(H)** Sample information.

TABLE S3 Summarized transcriptome data matrix and significantly changed transcripts obtained by microarrays. FC, fold change; FDR, false discovery rate.

TABLE S4 Summarized transcriptome data matrix and significantly changed transcripts obtained by RNA-Seq. FC, fold change; FDR, false discovery rate.

TABLE S5 Results of enrichment analysis. The top five gene ontology (GO) terms (full) that were significantly enriched (hypergeometric test with Benjamini and Hochberg FDR correction, FDR < 0.05) among differentially expressed genes (DEGs) whose expression differed significantly in response to HL (LIMMA, FDR < 0.05 and |log_2_FC|≥ 1) based on microarray **(A)** and RNA-Seq **(B)** analysis. FDR, false discovery rate; NS, not significant.
