# Supplementary material for: A Systems Analysis With “Simplified Source-Sink Model” Reveals Metabolic Reprogramming in a Pair of Source-to-Sink Organs During Early Fruit Development in Tomato by LED Light Treatments
Source: Front Plant Sci. 2018 Oct 9;9:1439. doi: 10.3389/fpls.2018.01439 (PMC6191670; doi:10.3389/fpls.2018.01439)
Supplement: FIGURE S1 — Simplified source-sink model and overall experimental design. (A) Schematic representation of red LED lighting in this study, named “simplified source-sink model.” We removed all the leaves and trusses except for the flowers of the second truss, the leaf just below the second truss, and the apical portions of the main shoot at the anthesis stage of the second truss. (B) Tomato plant cultivation with our systems under three types of light intensities (P200, P500, and P1000). [file Data_Sheet_1.pdf]

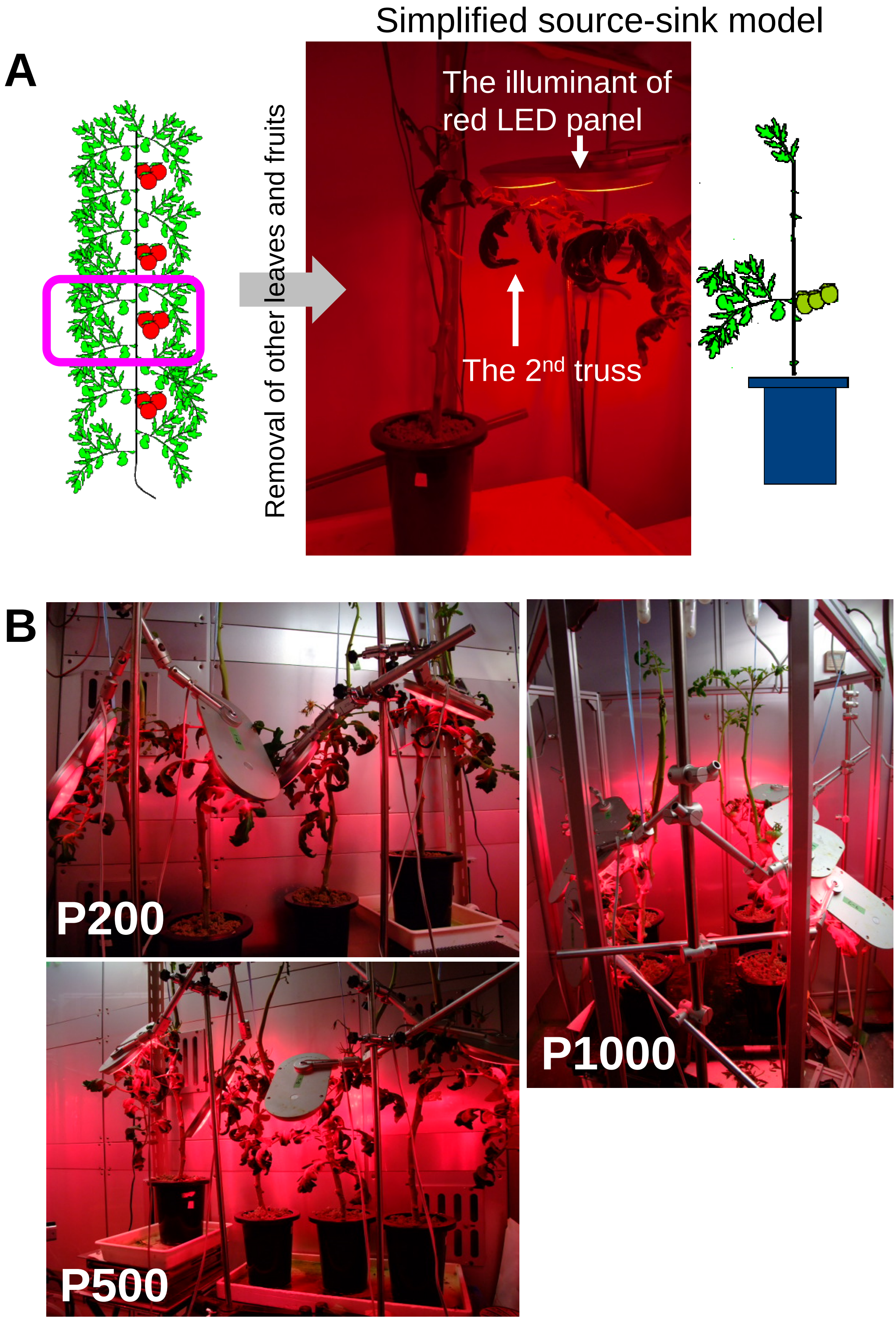

Fukushima et al. Supplemental Figure S1

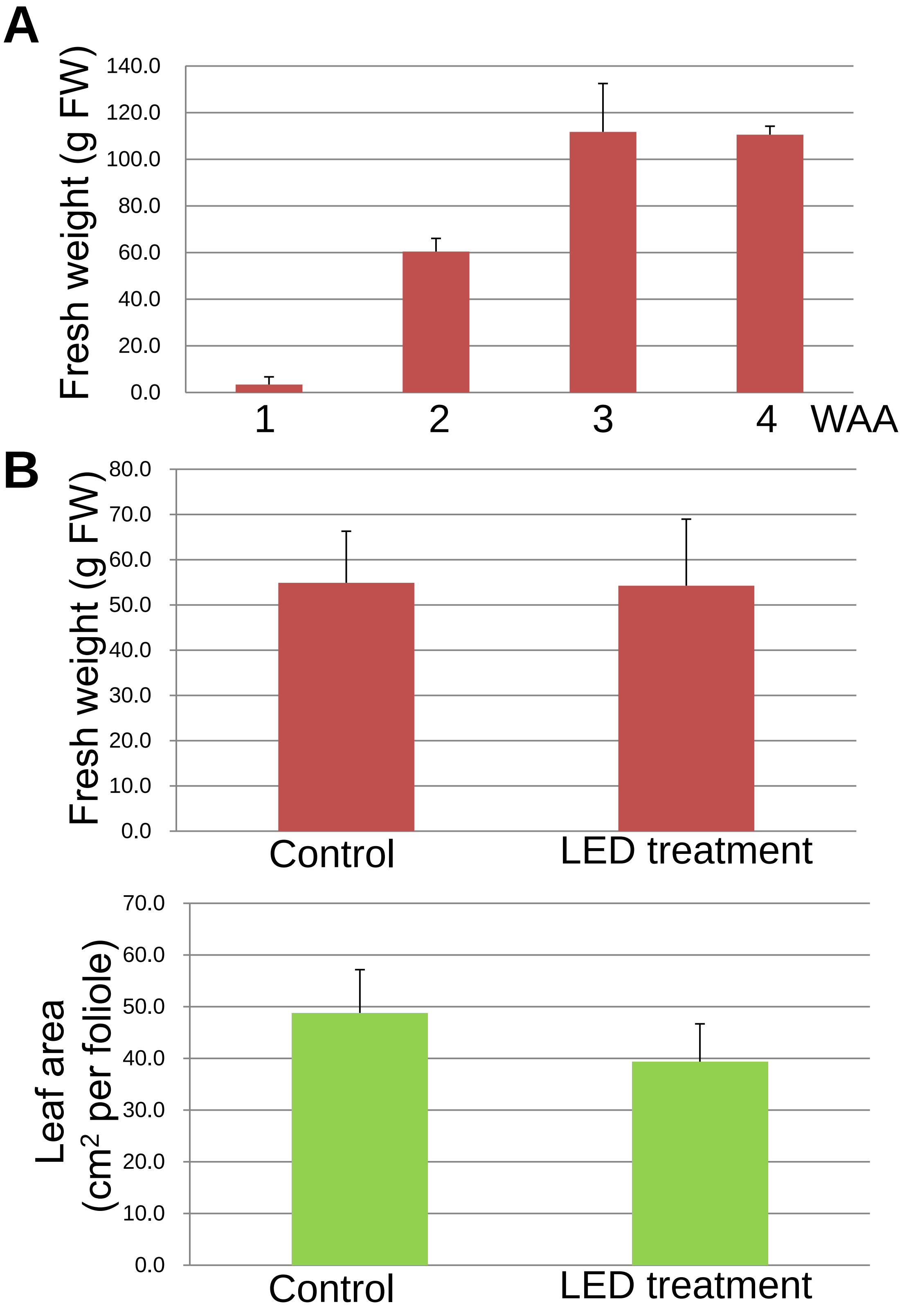

Fukushima et al. Supplemental Figure S2

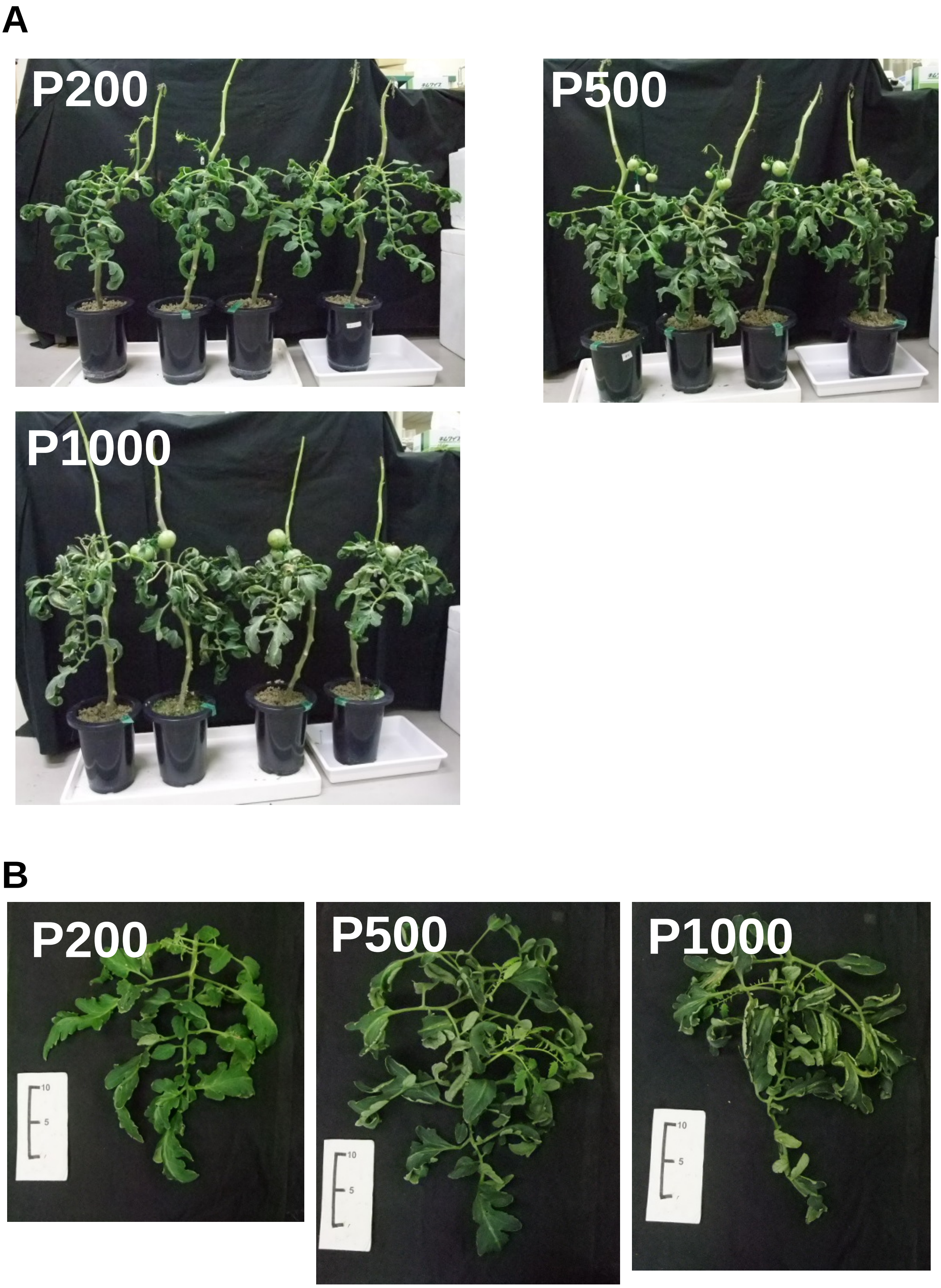

Fukushima et al. Supplemental Figure S3

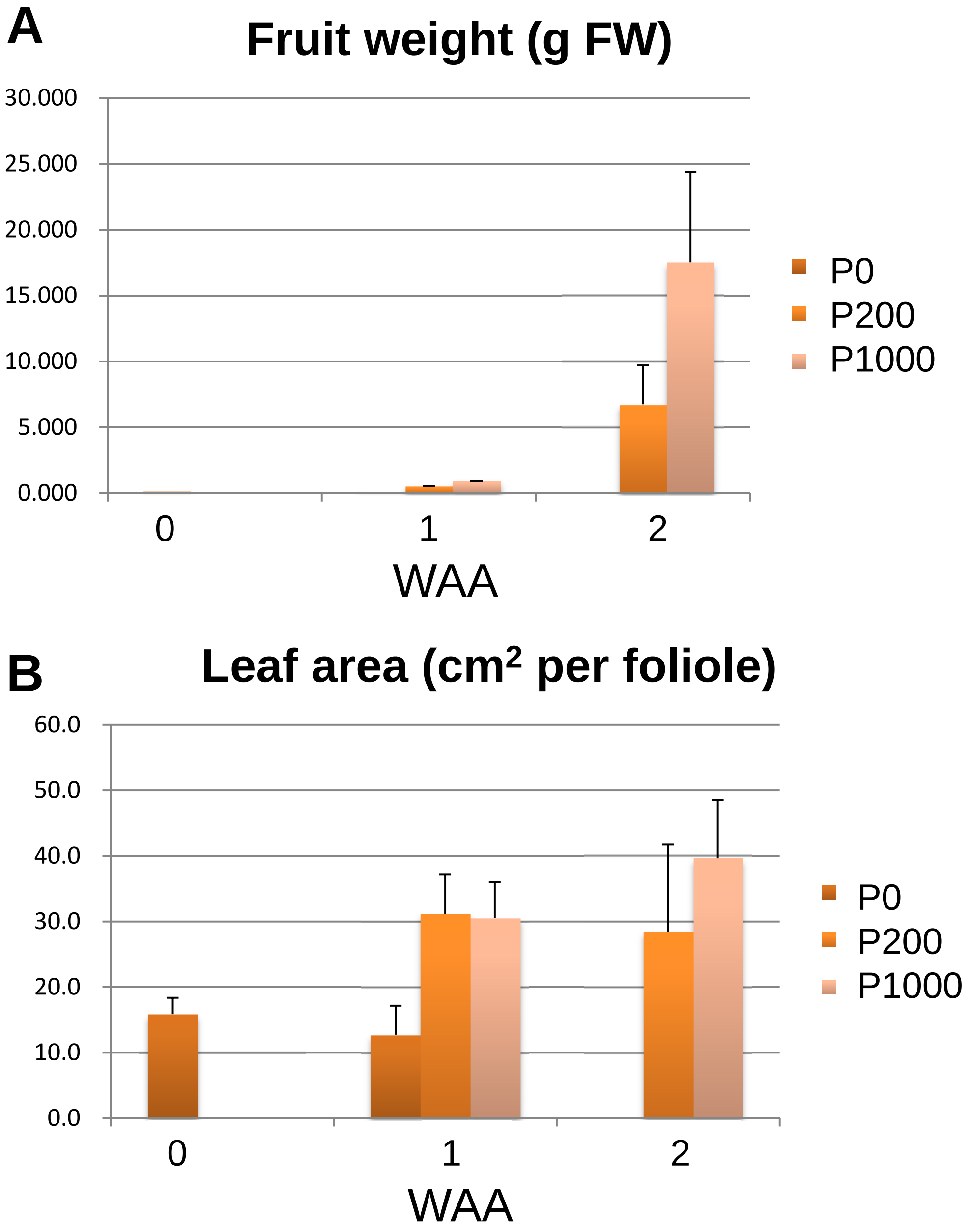

Fukushima et al. Supplemental Figure S4

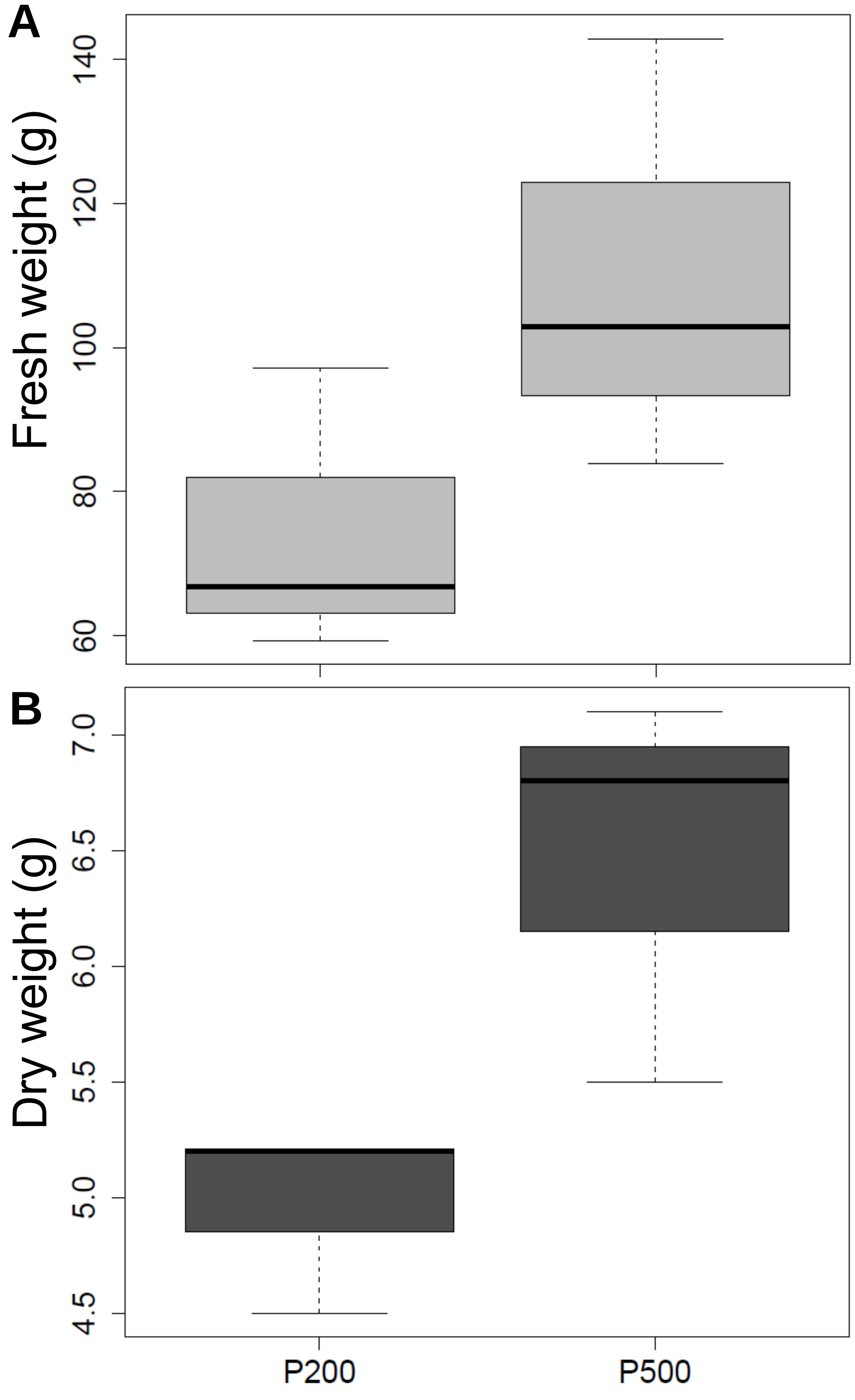

Fukushima et al. Supplemental Figure S5

Experimental Design for Metabolite Profiling

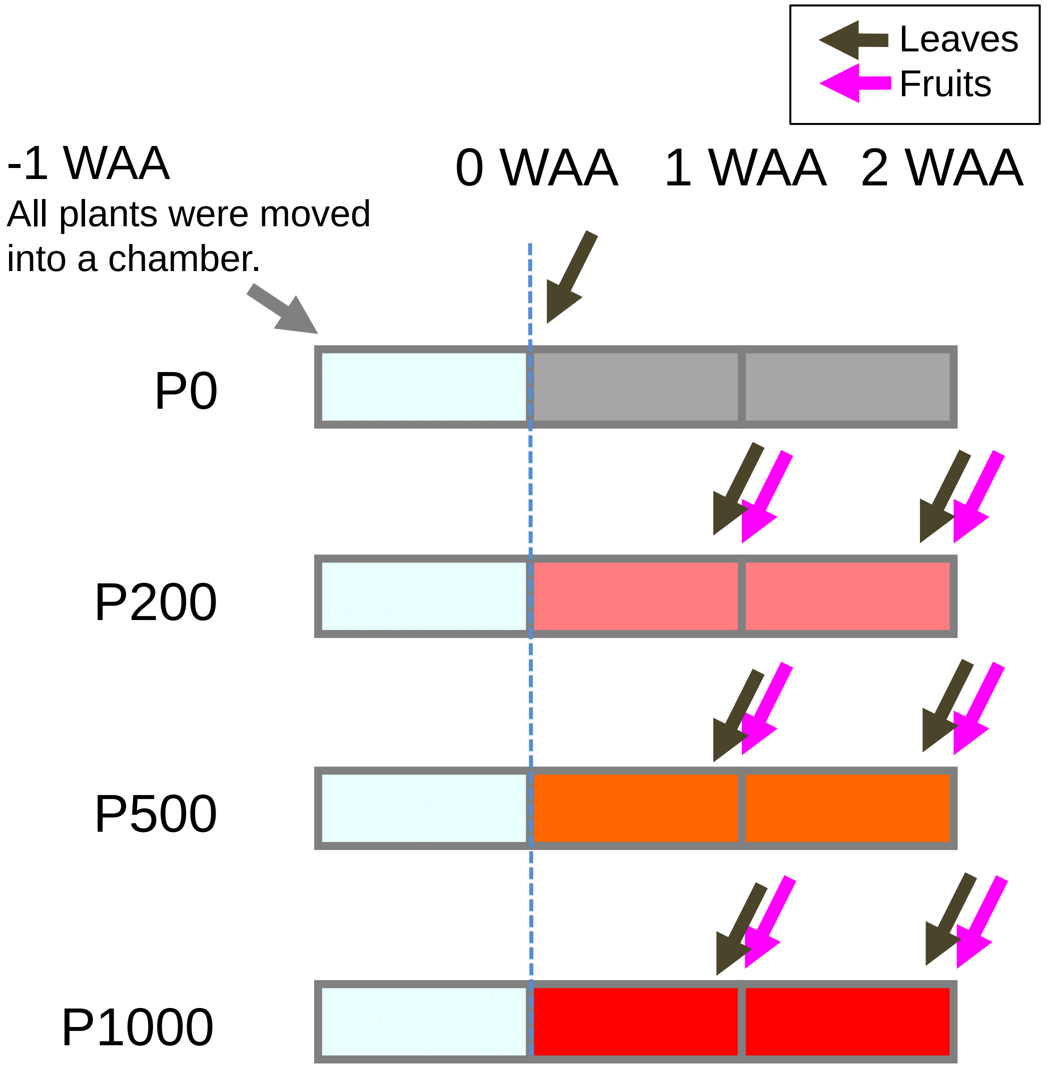

WAA, weeks after anthesis

Fukushima et al. Supplemental Figure S6

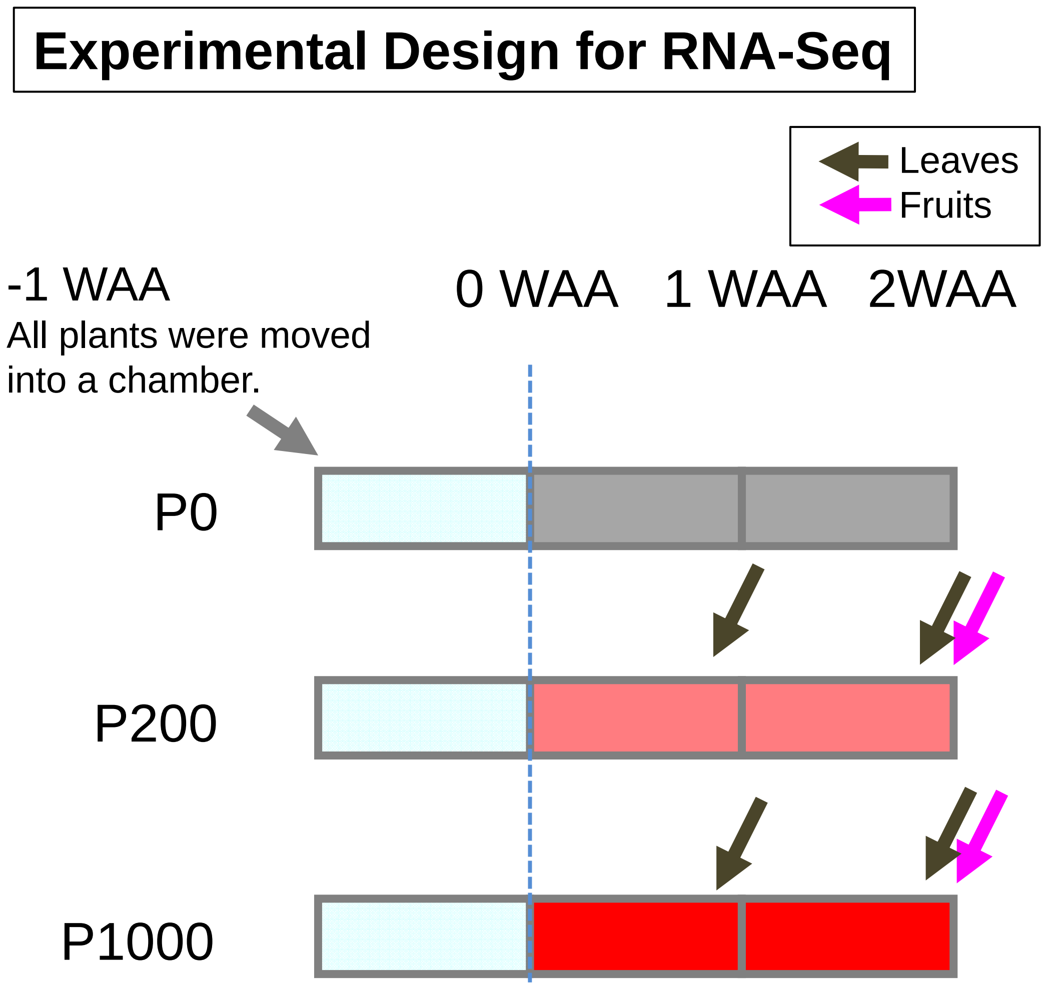

WAA, weeks after anthesis

Fukushima et al. Supplemental Figure S7

Experimental Design for microarray

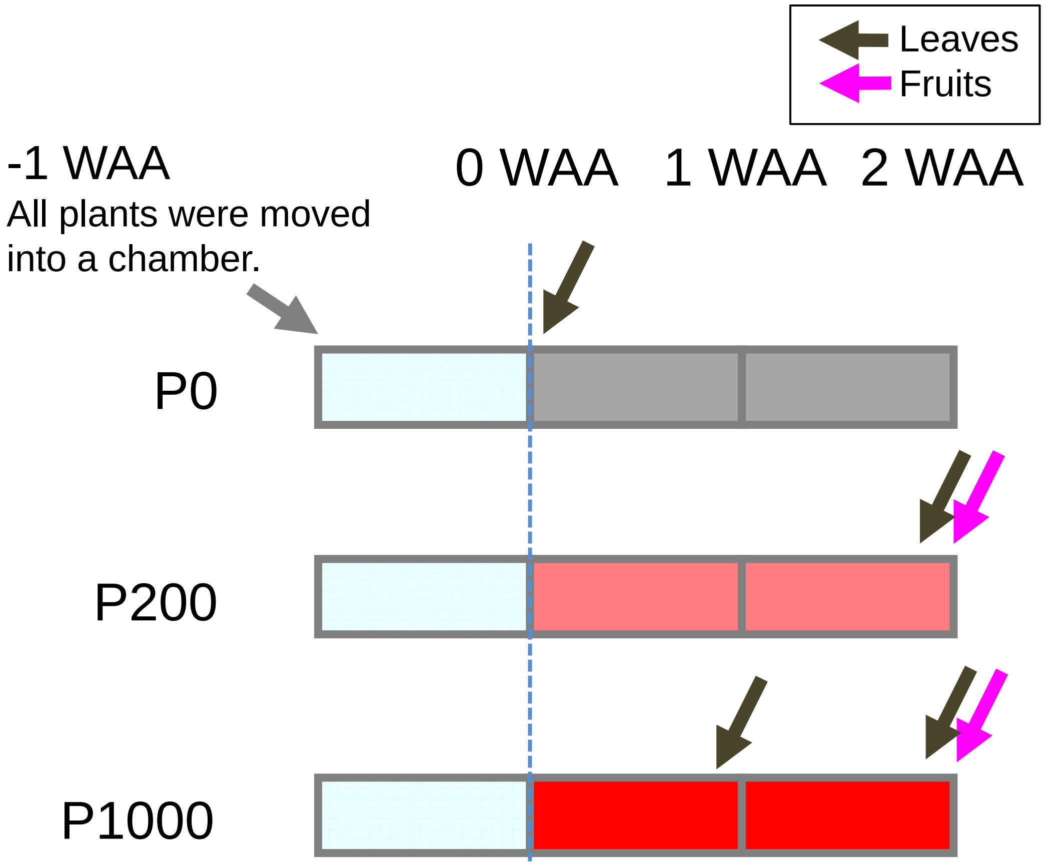

WAA, weeks after anthesis

Fukushima et al. Supplemental Figure S8

A. Summary of the sequence assembly with Illumina HiSeq 2000

Sample name	Total Reads	Total Nucleotides (nt)	Q20 %	N %	GC %
Leaf 1 WAA [P200]	26,822,224	2,414,000,160	**95.73**	0.01	42.89
Leaf 1 WAA [P1000]	26,822,224	2,414,000,160	**95.80**	0.01	43.51
Leaf 2 WAA [P200]	27,466,668	2,472,000,120	**96.87**	0.00	44.09
Leaf 2 WAA [P1000]	26,288,888	2,365,999,920	**96.78**	0.00	43.68
Fruit 2 WAA [P200]	21,308,372	1,917,753,480	**97.31**	0.02	44.70
Fruit 2 WAA [P1000]	24,483,526	2,203,517,340	**97.38**	0.02	44.47

B. Mapping statistics for each sample by Bowtie

	Leaf, 1 WAA [P200]	Leaf, 1 WAA [P1000]	Leaf, 2 WAA [P200]	Leaf, 2 WAA [P1000]	Fruit, 2 WAA [P200]	Fruit 2, WAA [P1000]
# reads processed	42,648,590	36,548,197	30,774,242	31,898,213	21,879,573	36,548,197
# reads with at least one reported alignment	22,331,303 (52.36%)	20,180,684 (55.22%)	19,690,785 (63.98%)	20,024,143 (62.78%)	14,368,079 (65.67%)	20,180,684 (55.22%)
# reads that failed to align	18,460,792 (43.29%)	14,812,210 (40.53%)	10,087,227 (32.78%)	10,873,750 (34.09%)	6,908,852 (31.58%)	14,812,210 (40.53%)
# reads with alignments suppressed due to -m	1,856,495 (4.35%)	1,555,303 (4.26%)	996,230 (3.24%)	1,000,320 (3.14%)	602,642 (2.75%)	1,555,303 (4.26%)

Fukushima et al. Supplemental Figure S9

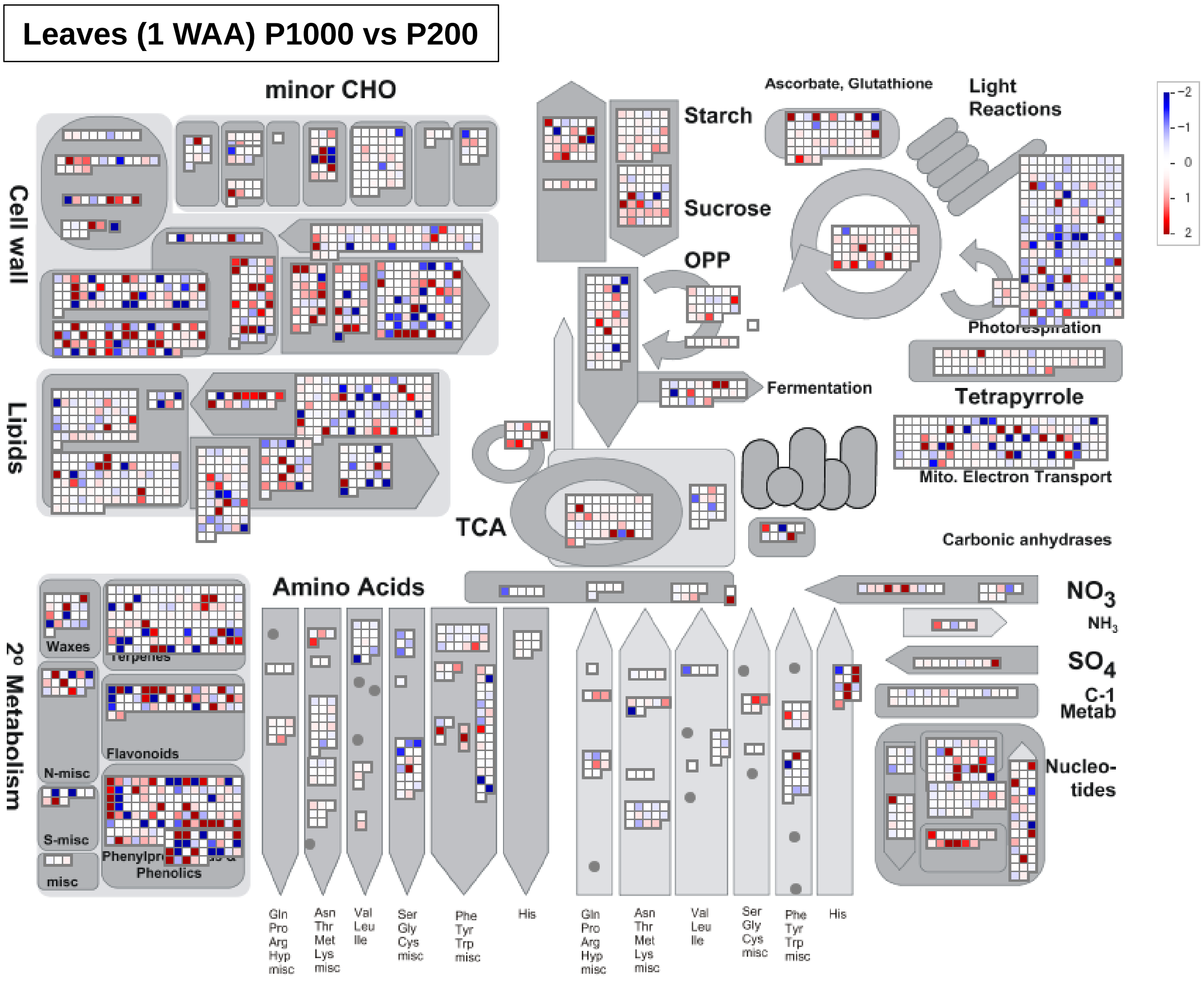

Fukushima et al. Supplemental Figure S10
